# Global Warming Implications of the Use of By-Products and Recycled Materials in Western Australia’s Housing Sector

**DOI:** 10.3390/ma8105347

**Published:** 2015-10-12

**Authors:** Krishna Lawania, Prabir Sarker, Wahidul Biswas

**Affiliations:** 1 Sustainable Engineering Group, Curtin University, Western Australia 6845, Australia; w.biswas@curtin.edu.au; 2 Department of Civil Engineering, Curtin University, Western Australia 6845, Australia; p.sarker@curtin.edu.au

**Keywords:** clay bricks, cast *in-situ* sandwich walls, fly ash, GGBFS, RCA, PET foam, GHG emissions

## Abstract

Western Australia’s housing sector is growing rapidly and around half a million houses are expected to be built by 2030, which not only will result in increased energy and resources demand but will have socio-economic impacts. Majority of Western Australians live in detached houses made of energy intensive clay bricks, which have a high potential to generate construction and demolition (C&D) waste. Therefore, there is a need to look into the use of alternative materials and construction methods. Due to Western Australia’s temperate climate, concrete could not only offer a comfortable living space but an operational energy saving also can be achieved. This paper has assessed the global warming implications of cast *in-situ* concrete sandwich wall system as an alternative to clay brick walls (CBW) with partial replacement of cement in concrete with by-products such as fly ash (FA) and ground granulated blast furnace slag (GGBFS), natural aggregate (NA) with recycled crushed aggregate (RCA), natural sand (NS) with manufactured sand (MS) and, polyethylene terephthalate (PET) foam core as a replacement to polystyrene core for construction of a typical 4 × 2 × 2 detached house in Perth. Life cycle management (LCM) approach has been used to determine global warming reduction benefits due to the use of available by-products and recycled materials in Western Australian houses.

## 1. Introduction

### 1.1. Sustainable Built Environment

Global warming impact, resource scarcity and waste generation are some predominant environmental impacts of modern civilization. Australia’s current per capita carbon footprint (23.1 tonnes of CO_2_ e-) and ecological footprint (6.3 global hectares) are approximately 5 and 3.5 times higher than the global average [1,2] mainly due to rapid population and economic growth [3].

Globally, the resource intensive building sector annually consumes 25% of the wood harvest, 40% of stone, sand and gravel, and 16% of water [4]. In order for building sector to achieve sustainability, the overall approach has to change from the use of non-renewable resources to use of renewable resources and from reduction of waste to reuse and recycling of waste [5]. The use of low impact, renewable and recyclable building materials and the reusability of industrial by-products for construction purposes should be prioritised in the design, construction and management to achieve environmentally sustainable infrastructure [6,7].

The building sector in Australia contributes to 20% and 23% of country’s annual energy and GHG emissions, respectively [8,9]. As a result, global warming impact and embodied energy consumption are commonly used key indicators to assess the environmental performance of building sector [10,11,12,13]. Australia’s housing sector is energy and carbon intensive because a majority of Australians prefer to live in detached houses made of energy intensive clay bricks [14,15] and due to rapid population growth, this sector is expected to grow rapidly from 8.7 million houses in 2010 to 12 million houses by 2030 [16]. During this period, about 15% (460,000 houses) of these new houses will be constructed in Western Australia alone. Additionally, the building sector in Australia is generating about 20 million tonnes of C&D wastes annually [17] of which the clay brick itself accounts for 16% [18] and these wastes contain huge amount of embodied energy [19]. Australian residential building sector’s GHG emissions are growing at 1.3% annually [8] which will impede Australia to meet 60% GHG emissions reduction target by 2050.

The strategies need to be developed for the reduction of energy intensive clay bricks and associated C&D wastes and two possible ways to attain these strategies are to design alternative wall systems requiring less materials and the replacement of virgin materials with recyclates.

It is therefore, imperative to develop an environmental tool that will enable engineers, developers and planners to estimate the GHG emissions and embodied energy saving benefits of the use of alternative wall systems and by-products. Life Cycle Assessment (LCA) tool has been utilized widely to assess the life cycle GHG emissions and embodied energy consumption of the infrastructure industries [10,13,20,21] and to identify further environmental improvement opportunities. This tool captures the overall environmental impacts of a product, process or services from mining, production, assembly, operation, to end of life and thus making the LCA a unique approach in the suite of environmental management tools [22,23].

This paper has considered the current practice of clay brick wall house (CBWH) in Perth as a case study. A LCM framework has been developed to reduce the GHG emissions and embodied energy consumption associated with the construction and use of CBWH.

Firstly, this paper presents GHG emissions and embodied energy consumption of CBWH in Perth, Western Australia. Secondly, GHG emission and embodied energy consumption saving potential associated with the use of alternative wall system have been estimated. Thirdly, the use of by-products and recyclates has been considered to further reduce the environmental impacts. Finally, some other indirect environmental benefits that will result from the reduction of virgin and energy intensive materials use have been estimated.

### 1.2. Life Cycle Management Framework

**Figure 1:**
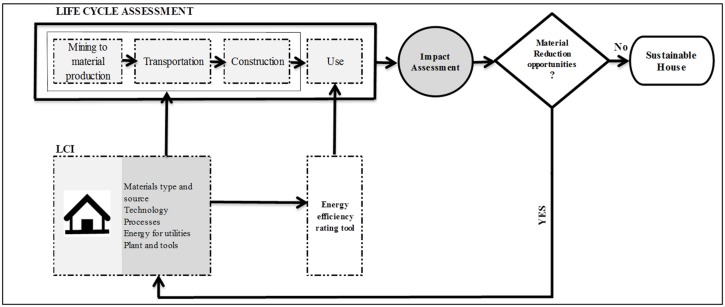
Life cycle management (LCM) framework.

The LCM framework for this study as shown in Figure 1, integrates LCA tool with the widely used industrial tool known as Nationwide House Energy Rating Scheme (NatHERS) tool [24,25,26] and strategies involving the utilization of less material intensive wall system, by-products and recyclates in housing sector in Western Australia. This LCM framework will enable to estimate the environmental implications of the use of by-products and recyclates in concrete walls in building construction. The information on materials and energy required during mining to material production, transportation of material to construction site, construction activities and use stages have been considered to conduct this LCA analysis. Since the demolition and disposal of wastes to landfill or their recycling or reuse of the demolition wastes are not considered, it is best termed as a streamlined LCA (SLCA) [10,27].

The life time of the house has been considered as 50 years following Islam and TBA [28,29]. The layout and design of a typical 4 × 2 × 2 (4 bedrooms, 2 bathrooms and 2 car parks) detached CBWH in Perth has been utilized to estimate the amount of materials and energy required during pre-construction and construction stages of building life. The energy required for heating, cooling, lighting and hot water over the life of the house has been calculated using NatHERS tool (V2.0.2.13SP2). The inputs required for all stages of building life have been estimated to develop a life cycle inventory (LCI), which is a pre-requisite for carrying out a life cycle assessment. The data from LCI have been inserted into SimaPro 8.02 LCA software to calculate GHG emissions and embodied energy consumption benefits associated with the reduction of virgin materials in houses. The use of less material intensive wall system and by-products and recyclates can substantially reduce material consumption and associated environmental impacts.

#### 1.2.1. Streamlined Life Cycle Assessment

This SLCA has employed the four steps of ISO 14040-44 [23,30]: (1) goal and scope definition; (2) inventory analysis; (3) impact assessment; and (4) interpretation (as presented in the “Results” section of this report) in order to calculate the global warming implications of the use of by products and recycled materials for the construction of houses in WA.

##### Goal and Scope

The goal is to assess the life cycle global warming and embodied energy consumption implications of the use of less material intensive wall system, by-products and recycled materials in residential buildings of Western Australia. The functional unit for this study is the construction and use of a typical 4 × 2 × 2 house. The system boundary for this study is limited up to use stage only.

Only GHG emissions and embodied energy consumption have been considered for this study as these are two predominant impacts resulting from the building sector as these impacts have been considered by other studies [10,13]. Other associated environmental impacts *i.e.*, acidification, eutrophication, human and eco-toxicity, are insignificant compared to aforementioned impacts [29]. The loose furniture, services, accessories and, external site developments have been excluded from this study as they are not linked to the performance of the building.

##### Life Cycle Inventory

A detailed LCI of a typical house in Perth consisting of detailed information on the Bill of Quantities, transportation of construction materials (tkm = tonne × km travelled), and energy required for plants and tools at site during construction stage and energy consumed during use stage were generated using detailed drawings, sources of materials, data sheets and AccuRate software. Table 1a,b show the life cycle inventory of this 4 × 2 × 2 detached CBWH.

Table 1(**a**) Materials used for the construction of the typical current practice of clay brick wall house (CBWH) in Perth; (**b**) Energy used for the construction stage of a typical CBWH in Perth.

**Table:** (**a**)

Sr. No.	Material	Location	Amount	Unit	Average Distance (km)	tkm
1	Virgin sand	Neerabup/The Lakes	21.15	m^3^	50	1797.75
2	Polythene sheet	Canning Vale	0.07	m^3^	30	1.31
3	Mesh & bar reinforcement	Bibra Lake	253.69	m^2^	30	19.03
4	Ready mix concrete	Canning Vale/Belmont	32.65	m^3^	30	2350.62
5	Clay bricks	Middle Swan/Hazelmere	48.52	m^3^	30	2838.42
6	Brickie sand	Neerabup/The Lakes	5.69	m^3^	50	484.05
7	Cement	Munster	2.63	tonne	30	78.75
8	Lime	Kwinana/Munster	0.91	tonne	30	27.20
9	Metal lintels	Malaga/Wangara	0.58	tonne	30	17.41
10	Aluminium windows	Belmont/Maddington	1.43	tonne	30	43.04
11	Metal door frames	Maddington	0.18	tonne	30	5.40
12	Roof Timber	Rockingham	7.50	m^3^	30	123.75
13	Roof Tiles	Middle Swan/Hazelmere	290.40	m^2^	30	435.60
14	Bat Insulation for Roof	Canning Vale	264.00	m^2^	30	14.26
15	Gyprock boards & cornices	Canning Vale	264.00	m^2^	30	59.40
16	Plaster sand	Neerabup/The Lakes	5.19	m^3^	50	440.76
17	Door shutters	Armadale/Bibra Lake	0.74	m^3^	30	11.14
18	Ceramic floor and wall tiles	Osborne Park/Fremantle	2.09	m^3^	30	184.58

**Table:** (**b**)

Main Activities	Sub-Activities	Energy	Unit
Excavation and compaction	Bobcat and roller compactor operation	9	Hrs.
Loading of excavated soil into trucks	Front end loader operation	3	Hrs.
Mortar Mixing for brick work and rendering	Electricity consumption by Mortar Mixer	60	kWh
Lifting of bricks/lintels/roof beams	Fork Lift operation	20	Hrs.
Cutting of bricks, timber, steel and tiles, drilling, nailing, grinding & sanding	Electricity consumption by Hand tools	60	kWh
Cart away of excavated soil and construction waste	Transport operation	3409	tkm

##### Impact Assessment

The GHG emissions and embodied energy consumption assessment of the house consists of two steps. In the first step, total GHG emissions and energy consumption in each process are calculated, and then in the second step they are converted to CO_2_ equivalent (CO_2_ e-) for GHG emissions impact and to GJ (giga joule) for embodied energy consumption.

*Step 1:* The LCI data were entered into SimaPro 8.02 [31] LCA software. Each input was linked to relevant library in the SimaPro 8.02 software. The libraries in this software contain the emission factors of energy, materials and transportation inputs for estimating the environmental impacts. The libraries of local products or Australian emission factor databases have been selected to represent local conditions. In the absence of local database in the software, new library databases have been created by obtaining the information on raw material and energy consumptions from local reports. When local information were unavailable for developing libraries of these materials, Ecoinvent Unit Process (EUP) libraries have been used for assessing GHG emissions [32].

*Step 2:* Once the inputs in the inventories have been linked to the relevant libraries in the software, the Australian GHG method was used to estimate the GHG emissions and the Cumulative Energy Demand method was used to determine the embodied energy consumption of CBWH in Perth including the breakdown of GHG emissions and embodied energy consumption to help identify the “hotspot(s)”.

#### 1.2.2. Application of Cleaner Production Strategies

Once the SLCA of a CBWH in Perth has been performed, Cleaner Production Strategies (CPS) have been applied to further reduce material consumption and associated environmental impacts. Cleaner production is an integral, necessary component for achieving sustainable development and it helps in increasing the productive use of natural resources, minimizing generation of waste and emissions and avoiding risk to people and communities [33,34]. The cleaner production initiatives are applied to processes, products and services to increase efficiency and reduce negative environmental impacts [35]. Two main CPS that are mainly helpful in the reduction of virgin materials consumption in buildings have been considered.

#### 1.2.3. Product Modification Cleaner Production Strategy

This strategy involves the replacement of clay brick walls by a less material intensive wall system. A number of wall systems such as autoclaved aerated concrete block masonry, pre-cast concrete wall panels, stud walls and sandwich wall panels that can potentially be used in Australian building sector [18] have been investigated. Because of its high thermal mass and material saving reasons, the currant analysis has considered cast *in-situ* concrete sandwich walls (CCSW) as a potential replacement of CBW. The CCSW system has been successfully used in Europe, Middle East and Asia, where its popularity is gradually increasing. Additionally, this wall system has successfully been trialled in the eastern states of Australia [36] and complied with the BCA (Building Code of Australia) requirements. However, no initiatives are being taken in WA to build houses using CCSW.

The CCSW system consists of a welded wire space frame integrated with an expanded polystyrene (EPS) insulation core with thin layers of concrete sprayed on either side through shotcrete process after placing in position [36]. This system provides combination of both lightweight and thermal mass, built-in insulation, resistance to earthquake and fire, low moisture absorption and constructability [37]. In addition, the structural efficacy of this system for different construction applications has been established through various analytical and experimental studies [38,39,40,41,42,43].

An external CCSW that consists of 100 mm insulation core sandwiched within two layers of 50 mm thick concrete on either faces and internal walls consists of 50 mm insulation core sandwiched within two layers of 40 mm thick concrete on either faces has satisfied the Building code of Australia (BCA) requirements [44]. All non-wall elements, fixtures and features remain the same as of a clay brick wall house.

Whilst the replacement of CBW with CCSW could reduce GHG emissions and embodied energy consumption, there are further opportunities for reducing these impacts by replacing constituents of concrete with by-products and recyclates. The use of by-products helps in converting the linear system of production into close loop system of production by avoiding quarrying activities for virgin material and by reducing landfill area.

#### 1.2.4. Recycling Strategy

The constituents of conventional cement concrete were partially substituted by combination of by-products including, fly ash (FA), ground granulated blast furnace slag (GGBFS), recycled crushed aggregates (RCA) and manufactured sand (MS) and the replacement of polystyrene core with polyethylene terephthalate (PET) foam manufactured from post consumed PET bottles for sandwich wall.

##### Substitution of Concrete Constituents with By-Products and Recyclates

Three main ingredients of concrete, including cement, aggregates and sand have been considered for replacement with by-products and recyclates to further reduce the amount of virgin materials.

As per Australian Cement Industry Federation report, the GHG emission intensity of per tonne cement manufacturing in 2012–2013 was 700 kg CO_2_ e- [45]. Therefore, there is a need for substitution of cement in concrete with supplementary cementitious materials (SCM) with the one with low carbon footprint while maintaining the structural performance and integrity. FA and GGBFS can potentially be used as SCM in a structurally sound manner for the production of concrete [46]. FA is a pozzolanic material, which hardens by reacting with the calcium hydroxide released during the hydration of portland cement whereas GGBFS react with water to form hardened binder in the presence of portland cement due to its latent hydraulicity [46].

The second major constituent of concrete is crushed rock aggregate involving energy intensive crushing processes. The use of RCA produced from C&D waste could not only help reduce the amount of GHG emissions associated with energy consumption and landfill area but it could reduce the air-borne CO_2_ emissions as well [47]. The third major constituent of concrete is fine aggregate or natural sand. The MS, which is a by-product of crushed rock aggregate, could partially substitute the scarce natural sand resources [47], thereby avoiding additional processing in relation to quarrying and transporting natural sand. Whilst the conversion of MS to workable construction material would require processing; it is worth investigating into the environmental benefits associated with the replacement of natural sand with MS.

##### Structural and Environmental Implications of By-Products and Recyclates in Concrete

Table 2, show the percentage range of existing constituents that can potentially be replaced with by-products and recyclates, and have also been successfully trialed to study the mechanical and structural behavior and performance, such as compressive and tensile strength, permeability, shrinkage, and durability.

Flower and Sanjayan, Malhotra, Mehta and Dhir (as cited by O’Brien) [48] suggested that the substitution of FA with Portland cement reduces the embodied GHG emissions of concrete. Nath and Sarker [49,50] suggested that 30%–40% cement can be replaced with FA for high strength concrete and a further adjustment in the concrete mix can increase strength, reduce shrinkage and improve permeability properties. For high strength concrete, the risk of thermal cracking can be avoided by replacing cement with 40% FA [51]. Berndt [52] investigated various properties such as compressive and tensile strengths, elastic modulus, coefficient of permeability and durability in chloride and sulphate solutions of 40MPa concrete mixes having no cement substitution, 50% cement replaced with FA, 50% and 70% cement replaced with GGBFS, and 50% cement replaced equally with FA and GGBFS, and all mixes with natural and recycled aggregates. The concrete mixes containing 50% GGBFS have the best overall mechanical properties and durability for both natural aggregate and RCA [52]. In a 25 MPa concrete mix, where FA is combined with RCA, it not only improves the mechanical properties, and durability performance but helps to overcome negative effects associated with the use of RCA [53].

**Table 2:** Percentage replacements of concrete constituents considered for various studies.

Concrete Grade	Cement Substitution		Aggregate Substitution	Sand Substitution	References
	FA	GGBFS	RCA	MS	
40 MPa	25%–50%	25%–70%			Berndt, 2009 [52]
40 MPa	30%	50%–80%			Elchalakani *et al.*, 2014 [54]
35 MPa		20%–40%			Arivalagam, 2014 [55]
50 MPa	30%–40%				Nath and Sarker, 2011 [50]
62–68 MPa	30%–40%				Sarker and Mckenzie, 2009 [51]
25–32 MPa	25%	40%			Flower and Sanjayan, 2007 [56]
32 MPa		70%			Crossin, 2015 [57]
30 MPa			65%		Marinkovic *et al.*, 2010 [58]
40 MPa	40%		25%–100%		Ahmed, 2012 [59]
30–35 MPa	30%		70%–85%		Corinaldesi and Moriconi, 2009 [60]
30–60 MPa				100%	Chow *et al.*, 2013 [61]
30 MPa				20%–80%	Naveenth and Satheeshkumar, 2015 [62]
20 MPa				20%–100%	Jadhav and Kulkarni, 2012 [63]
50 MPa				30%–100%	Sheng-Dong *et al.*, 2015 [64]
60 MPa	35%	55%	50%–100%		Kou *et al.*, 2011 [65]
25 MPa			25%–100%	35%–45%	Etxeberria *et al.*, 2010 [66]
20 MPa, 45 MPa, 65 MPa			100%		D Pedro *et al.*, 2014 [67]

The physical properties of RCA can only enable it to be applied in low-to-middle strength structural concrete [58,68]. On the other hand, FA aggregate that is obtained by sintering and crushing process and slag aggregate has not only been found to be lighter and stronger than natural aggregate but also possesses lower thermal conductivity [69,70]. Though, the increase of RCA may lower the 28 days compressive strength of concrete, but after a period of time, the strength will be higher than that of concrete with NA [71].

MS does not contain organic impurities and, its use in concrete reduces the demand for water and super plasticizer due to presence of microfines and increases the strength without changing the portion of cement in concrete [61,64,72]. Additionally, the blending of MS with natural sands improve workability of concrete and is effective in reducing the level of microfines in the fine aggregate used in the concrete mix as opposed to microfines in MS [73]. By substituting 20% to 60% of natural sand by MS in concrete, the strength of concrete increases proportionately [63,74] and optimum strength may be achieved with 40%–60% substitution [62,64].

Australia produced 12.3 million tonnes of fly ash in 2013 out of which 52% was utilized and in addition to new ash being produced every year there is stock of more than 400 million tonnes of fly ash [75]. As per Australasian (Iron & Steel) Slag Association report, approximately 1.3 million tonnes of slag was produced in year 2009 and the slag will remain available in substantial amount as long as iron is produced in Australia [76]. In year 2010, Australia produced around 1.3 million tonnes of recycled aggregate [77]. Approximately 30% of the quarry production is crushed fine, which can further be processed to obtain manufactured sand suitable for concrete [73].

**Figure 2:**
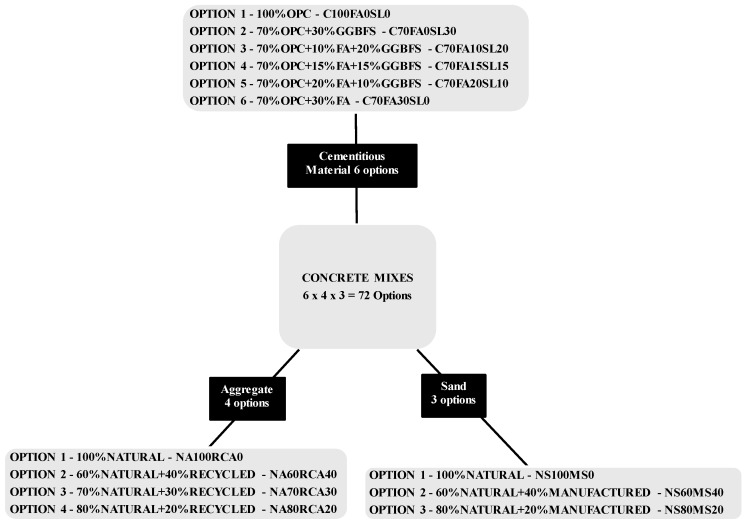
Concrete mixes with varying % of substitutions.

On the basis of the above mentioned findings, following conclusions were drawn for developing compositions of concrete utilizing FA, GGBFS, RCA and MS with conventional materials:

- Up to 30% of cementitious material and 40% of natural aggregate (NA) and 40% of natural sand (NS) could be substituted with FA, GGBFS, RCA and MS with minor adjustments in concrete mix design for various combinations with acceptable structural performance.
- The changes in market and products may affect the constant supply of by-products and therefore it is important to consider a number of structurally sound compositions to suit to any situation during the time of resource scarcity. As a result, seventy two possible compositions (one current/conventional + seventy one alternatives) of concrete have been considered for determination of possible GHG emissions saving implications of this research.
- The cementitious material is divided in to six groups as 100% OPC, 70% OPC + 30% FA, 70% OPC + 20% FA + 10% GGBFS, 70% OPC + 15% FA + 15% GGBFS, 70% OPC + 10% FA + 20% GGBFS, and 70% OPC + 30% GGBFS. Each of these cementitious composition has been used for four aggregate compositions including 100% NA, 60% NA + 40% RCA, 70% NA + 30% RCA, and 80% NA + 20% RCA, where each of these aggregate compositions are further sub-divided in to three sand categories each as 100% NS, 60% NS + 40% MS, and 80% NS + 20% MS each respectively. Therefore, the total number of compositions of concrete considered in this study is (6 × 4 × 3) or 72 as shown in Figure 2.

Accordingly, 72 different LCIs have been developed for determining the GHG emissions and embodied energy consumption of 72 compositions of concrete for construction of a CCSWH in Perth.

Once aforementioned CPS have been incorporated into LCI, SLCA has been carried out to estimate the amount of life cycle GHG emissions and embodied energy consumption that can be avoided by using recyclates, sandwich wall and by-products.

## 2. Results and Discussion

### 2.1. GHG Emission and Embodied Energy Consumption Assessment of WA’s Current Building Practice

**Figure 3:**
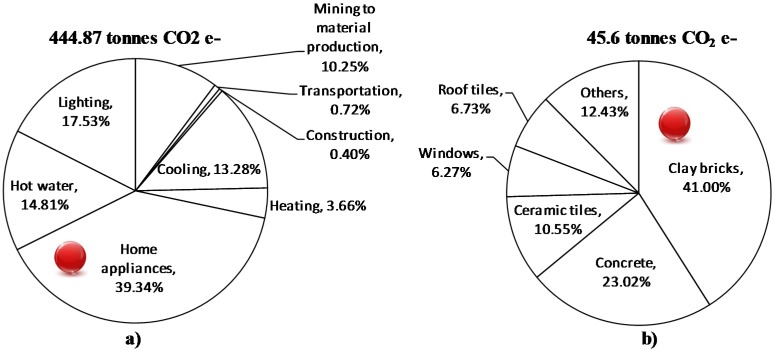
Breakdown of GHG emissions in terms of inputs (**a**) mining to use (**b**) mining to material production stage only.

The results of SLCA show that life cycle GHG emissions from mining to material production, transportation, construction, and usage stages for a typical CBWH of 243 m^2^ area are 444.87 tonnes CO_2_ e-. Figure 3a shows the breakdown of GHG emissions in terms of stages wherein home appliances that accounted for the largest share (39.34%) of the total GHG emissions have been considered to be the main hotspot. The wall and non-wall elements (pre-construction and construction activities) also contribute significant portion of GHG emissions (11.37%). Clay bricks (41%), and concrete (23.02%) have been found to be the top two carbon intensive materials during mining to material production stage (Figure 3b). All other materials such as ceramic tiles, doors, windows, steel, timber, roof tiles, and mortar together contributed to the remaining GHG emissions (*i.e.*, 35%).

**Figure 4:**
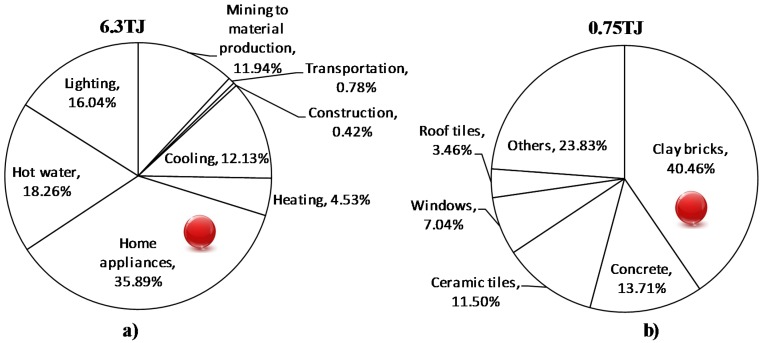
Breakdown of embodied energy consumption in terms of inputs: (**a**) mining to use (**b**) mining to material production stage.

The results of SLCA show that life cycle embodied energy consumption from mining to material production, transportation, construction, and usage stages for a typical CBWH of 243 m^2^ area is 6.3 TJ. Similar to GHG emissions trends, the home appliances with a share of 35.89% of embodied energy consumption have been considered as main hotspot. The share of wall and non-wall elements (pre-construction and construction activities) is 13.14% (Figure 4a). Clay bricks (40.46%) have been found to be the most energy intensive construction material (Figure 4b).

Thus, cast *in-situ* sandwich wall house (CCSWH) has been considered that will not only avoid the use of carbon intensive clay bricks, it will also increase the thermal performance of the existing buildings.

### 2.2. GHG Emissions and Embodied Energy Consumption Implications of the Use of Cast In-Situ Sandwich Wall Systems

Another SLCA analysis has been carried out for cast *in-situ* sandwich wall house (CCSWH) by taking material saving and thermal performance changes into account while maintaining shape and size of typical CBWH. The LCI data show that the energy required for heating and cooling can potentially be reduced by 58.45% and 18.73% respectively and also the material consumption can be reduced by 18.16% (47.47 tonnes). The results of SLCA as presented in Table 3 show that the life cycle GHG emissions and embodied energy consumption from mining to material production, transportation, construction, and usage stages for a typical CCSWH are 415.81 tonnes CO_2_ e- and 5.84TJ, respectively. Therefore, the replacement of CBW with CCSW could reduce the GHG emissions and embodied energy consumption by 5.8% and 5.5%, respectively. Additionally, 51.1%, 21.83%, 14.35%, 20.25%, and 36.36% of the GHG emission can be reduced from heating, cooling, mining to material production, transportation, and construction stages respectively. This replacement has no GHG emissions implications associated with the use of home appliances, lighting, and hot water as these appliances were assumed to be unchanged. Further improvement of CCSWH can be considered by incorporating by-products and recyclates in concrete.

The embodied energy consumption also follows similar reduction trends as the saving associated with heating, cooling, mining to material production, transportation, and construction stages are 50.88%, 21.86%, 15.98%, 20.08%, and 36.74% respectively. The reason for this reduction is mainly due to the avoidance of energy intensive clay bricks and increased thermal performance of the envelope. Table 3 shows that GHG emissions from home appliances still accounts for the largest share (42.09%) of the total GHG emissions and have been identified as the main hotspot. Similar to GHG emissions trends, the home appliances with a share of 35.89% of embodied energy consumption have been considered as main hotspot but the main focus of this study is to reduce environmental impacts through material of construction.

**Table 3:** Breakdown and comparison of GHG emissions and embodied energy consumption in terms of inputs for CBWH and cast *in-situ* sandwich wall house (CCSWH).

Stage	GHG Emissions (tonnes CO_2_ e-)			Embodied Energy (TJ)		
	CBWH	CCSWH	% Saving	CBWH	CCSWH	% Saving
Mining to material production	45.60	39.06	14.35%	0.75	0.63	15.98%
Transportation	3.21	2.56	20.25%	0.05	0.04	20.08%
Construction	1.76	1.12	36.36%	0.03	0.02	36.74%
Cooling	59.10	46.20	21.83%	0.76	0.60	21.86%
Heating	16.30	7.97	51.10%	0.29	0.14	50.88%
Home appliances	175.00	175.00	0.00%	2.26	2.26	0.00%
Hot water	65.90	65.90	0.00%	1.15	1.15	0.00%
Lighting	78.00	78.00	0.00%	1.01	1.01	0.00%
**Total**	**444.87**	**415.81**	**6.53%**	**6.30**	**5.84**	**7.17%**

### 2.3. GHG Emissions and Embodied Energy Consumption Implications of the Use of by-Products and Recyclates

**Figure 5:**
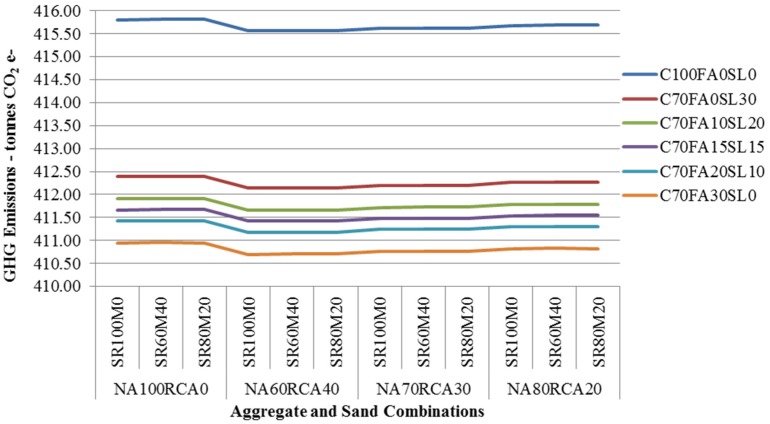
GHG Emissions of CCSWH using by-products for concrete.

**Figure 6:**
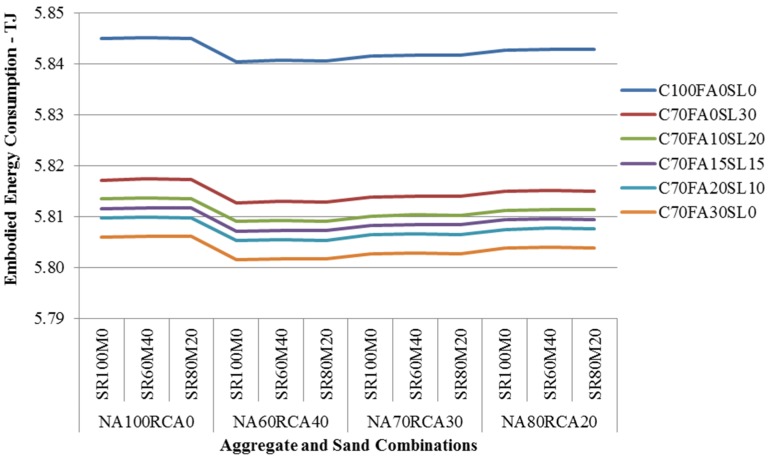
Embodied Energy consumption of CCSWH using by-products for concrete.

The LCI of CCSW has been modified to assess the environmental impacts of concrete made of different percentages of FA, GGBFS, RCA, MS and conventional materials. The results of SLCA shows that the use of by-product and recyclates in concrete can save a maximum of 25.91% (5.11tonnes CO_2_ e-) GHG emissions (Figure 5) and a maximum of 23.44% (43.34 GJ) embodied energy consumption (Figure 6) in comparison to the conventional concrete mix during the life cycle of the house. Similarly, a comparative LCA study of 1 m^3^ of various 40 MPa concrete mixes using combination of supplementary cementitious material and aggregates that was carried out by RMIT shows that the GHG emissions and embodied energy consumption of concrete utilizing supplementary cementitious material and aggregates are 12%–49% and 10%–42% lower than the conventional concrete of same grade respectively [78]. O’Brien *et al.* [48] suggested that even if FA is transported up to 11,000 km by articulated truck, 47,000 km by rail and 54,000 km by sea for replacing cement in concrete, the net GHG emissions will still be lower than that of conventional concrete. Therefore, the results of this study are within the range of similar studies conducted in Australia.

From the analysis of results, it is observed that the concrete with a composition of 70% OPC + 30% FA + 60% NA + 40% RCA + 100% NS could offer maximum amount of GHG emissions and embodied energy reduction. Even though, GGBFS and FA both are by-products but FA has slightly higher GHG emissions reduction potential because additional energy is consumed for grinding of granulated blast furnace slag. For 100% OPC concrete mixes, the changes in aggregate and sand compositions have minor impact on emissions. However, as the SCM are introduced, the GHG emissions and embodied energy consumption reduced significantly. This clearly shows that cement is highly energy intensive component of the concrete. An insignificant increase in GHG emissions (0.01% to 0.03%) and embodied energy consumption (0.05% to 0.14%) are observed when NS is partially substituted with MS in concrete mixes for all cementitious and aggregate compositions. The reason for this increase is due to the fact that an additional energy is consumed for making MS suitable for concrete, [79]. However, there are benefits from minimizing the waste and natural resource depletion [58].

Further analysis of results shows that the concrete mixes having aggregate composition of 60% NA + 40% RCA have higher GHG emissions and embodied energy consumption reduction potential while mixes having 80% NA + 20% RCA compositions have lower potential. Table 4 shows the GHG emissions and embodied energy consumption mitigation potential based on average values for each cementitious group.

**Table 4:** Concrete mix wise GHG emissions and embodied energy consumption mitigation potential.

Concrete Mix Compositions with All Aggregate Groups and Sand sub-Groups	% Mitigation Potential	
	GHG	Embodied Energy
Conventional concrete mix with 100% OPC	-	-
Concrete mix with 70% OPC and 30% GGBFS	17.47%	15.19%
Concrete mix with 70% OPC, 10% FA and 20% GGBFS	19.92%	17.23%
Concrete mix with 70% OPC, 15% FA and 15% GGBFS	21.15%	18.26%
Concrete mix with 70% OPC, 20% FA and 10% GGBFS	22.37%	19.28%
Concrete mix with 70% OPC, 30% FA	24.82%	21.32%

The concrete mixes having 30% cement substituted with FA provide maximum mitigation opportunity but other combinations with GGBFS also contribute to substantial reduction opportunity. Considering the availability constraints of these by-products, the above combinations offer a flexible solution.

Post consumed plastic bottles made of polyethylene terephthalate (PET) can be used either as a constituent of concrete or as a replacement of polystyrene core [80,81,82]. In normal conditions, PET is a non-degradable material and due to its large molecules, known micro-organisms are unable to consume it [83]. Sonia [84] suggested that recycling of PET into foam structure provides a durable insulation and core for sandwich structures. As per Australian national plastics recycling survey in 2013–2014, the recovery and recycling rate of PET was 54.8% [85]. Therefore, the use of post consumed plastic bottles in WA building industries could potentially increase the recovery rate and reduce the generation of solid wastes.

Recycled PET foam is considered as a replacement for polystyrene core for CCSW. The thermal performance of PET foam has been considered while calculating the heating and cooling energy using an AccuRate tool. SLCA analysis has been repeated for CCSWH with PET foam as core. The LCI data show that the energy required for heating and cooling of CCSWH could be further reduced by 7.87% and 2.02% respectively. The material consumption is marginally increased by 0.57% (1.22 tonnes). The results of SLCA as presented in Table 5 show that life cycle GHG emissions and embodied energy consumption from mining to material production, transportation, construction, and usage stages for a typical CCSWH that uses PET as core have been estimated to be 413.95 tonnes CO_2_ e- and 5.79 TJ, respectively. Therefore, the replacement of polystyrene core with PET core could reduce the GHG emissions and embodied energy consumption by 0.45% and 0.99%, respectively. Additionally, 7.78%, 1.95%, and 0.91% of the GHG emission can be reduced from heating, cooling, and mining to material production stages respectively. This replacement has no impact on GHG emissions associated with home appliances, lighting, and hot water. The GHG emissions associated with the transportation increased by 0.78%, which is negligible. The main reason for this increase is due to the fact that PET foam has higher density than polystyrene core. The embodied energy consumption also follows similar reduction trends as the saving associated with heating, cooling, and mining to material production are 7.86%, 1.84%, and 5.67% respectively.

**Table 5:** Breakdown and comparison of GHG emissions and embodied energy consumption in terms of inputs for CCSWH, and CCSWH with PET core.

Stage	GHG Emissions (Tonnes CO_2_ e-)			Embodied Energy (TJ)		
	CCSWH	CCSWH–PET	% Saving	CCSWH	CCSWH–PET	% Saving
Mining to material production	39.06	38.70	0.91%	0.63	0.60	5.67%
Transportation	2.56	2.58	-0.78%	0.04	0.04	-0.51%
Construction	1.12	1.12	0.00%	0.02	0.02	0.00%
Cooling	46.20	45.30	1.95%	0.60	0.59	1.84%
Heating	7.97	7.35	7.78%	0.14	0.13	7.86%
Home appliances	175.00	175.00	0.00%	2.26	2.26	0.00%
Hot water	65.90	65.90	0.00%	1.15	1.15	0.00%
Lighting	78.00	78.00	0.00%	1.01	1.01	0.00%
**Total**	**415.81**	**413.95**	**0.45%**	**5.84**	**5.79**	**0.99%**

The implementation of this strategy could reduce GHG emissions by 1.86 tonnes CO_2_ e- per house and embodied energy consumption by 0.06 TJ per house.

## 3. Summary of GHG and Embodied Energy Savings Due to Use of CPS

The results of implications of three cleaner production strategies comprising of incorporation of cast *in-situ* sandwich walls (product modification), partial replacement of cement with FA and GGBFS, NA with RCA and NS with MS (input substitution), and the use of PET foam made of post consumed recycled PET bottles (reuse and recycling) are presented in Table 6.

It appears from Table 6 that the implementation of product modification, input substitution and re-use and recycle strategies offers GHG emissions reduction of 6.53%, 1.15% and 0.42% respectively. Similarly, the implementation of product modification, input substitution and re-use and recycle strategies offers embodied energy consumption saving of 7.30%, 0.68% and 0.92% respectively. The overall GHG emissions reduction is 36.03 tonnes CO_2_ e- (8.10%) and embodied energy consumption saving of 0.56 TJ (8.90%) per house.

**Table 6:** Summary of mitigation potential after implementation of cleaner production strategies (CPS).

Impact	Original	Mitigation Potential Using CPS			Revised
		Product Modification	Input Substitution	Re-Use and Recycle	
GHG emissions–tonnes CO_2_ e-	*444.87*	29.06	5.11	1.86	**408.84**
Embodied Energy–TJ	*6.30*	0.46	0.04	0.06	**5.74**

## 4. Conclusions

The replacement of clay brick walls with cast *in-situ* sandwich walls for construction of house in Perth as well as subsequent implementation of CPS offers a GHG emissions reduction up to 36.03 tonnes CO_2_ e- and embodied energy consumption saving up to 0.56 TJ. The use of by-products is not only able to reduce the environmental impacts but it helps in minimizing the natural resource depletion and requirement of landfill area. Considering the growing demand for large number of houses in Perth, current approach could significantly reduce global warming impacts associated with the construction and use of clay brick houses and building industry can contribute to Australia’s commitment of GHG emissions reduction targets by 2050.
